# Effect of Vitamin B12 Supplementation on Depression: A Systematic Review of Randomized Controlled Trials

**DOI:** 10.1192/j.eurpsy.2026.11385

**Published:** 2026-07-31

**Authors:** M. F. Zeilstra, M. Arts, K. M. Zeilstra-Kalt, L. de Jonge

**Affiliations:** 1 Integrative Medicine, Center de Wetering, Leeuwarden; 2 Department of Geriatric Psychiatry and Neuropsychiatry, GGZ-WNB, Halsteren; 3 Integrative Medicine, Leonardo Scientific Research Institute, Groningen, Netherlands

## Abstract

**Introduction:**

Depression is a common psychiatric disorder with a multifactorial etiology. Emerging evidence suggests that nutritional deficiencies, particularly vitamin B12, may serve as modifiable risk factors for depressive symptoms. Vitamin B12 is essential for neurotransmitter synthesis, myelin formation, and homocysteine metabolism, all of which are implicated in mood regulation.

**Objectives:**

To systematically review randomized controlled trials (RCTs) evaluating the effects of vitamin B12 supplementation on depressive symptoms in adults.

**Methods:**

A systematic search of PubMed, Embase, and PsycINFO was conducted for RCTs published up to 2025. Eligible studies included adults diagnosed with depression or exhibiting elevated depressive symptoms who received vitamin B12 supplementation, either alone or in combination with standard treatments. Primary outcomes were changes in validated depression rating scales. Secondary outcomes included serum B12 levels, cognitive function, and adverse events. Extracted data were analyzed to assess efficacy, methodological quality, and consistency across studies.

**Results:**

Evidence indicates that vitamin B12 supplementation, particularly in individuals with low baseline B12 levels, may lead to meaningful reductions in depressive symptoms. Adjunctive therapy with antidepressants appears to further enhance treatment response. These effects are likely mediated by the normalization of homocysteine levels and the support of monoamine neurotransmitter synthesis. However, heterogeneity in sample sizes, supplementation dosages, and study durations limits comparability and generalizability.

**Conclusions:**

Current RCT evidence supports the potential of vitamin B12 supplementation as an adjunctive intervention in managing depression, especially among B12-deficient individuals. Larger, rigorously designed RCTs with standardized protocols are warranted to establish efficacy, optimal dosing, and long-term outcomes.

**Disclosure of Interest:**

None Declared

